# Absorption, metabolism and excretion of [^14^C]pomalidomide in humans following oral administration

**DOI:** 10.1007/s00280-012-2040-6

**Published:** 2012-12-01

**Authors:** Matthew Hoffmann, Claudia Kasserra, Josephine Reyes, Peter Schafer, Jolanta Kosek, Lori Capone, Anastasia Parton, Heasook Kim-Kang, Sekhar Surapaneni, Gondi Kumar

**Affiliations:** 1 Drug Metabolism and Pharmacokinetics Department, Celgene, 86 Morris Avenue, Summit, NJ 07901 USA; 2 Translational Development Department, Celgene, 86 Morris Avenue, Summit, NJ 07901 USA; 3 Clinical Pharmacology Department, Celgene, 86 Morris Avenue, Summit, NJ 07901 USA; 4 XenoBiotic Laboratories, Plainsboro, NJ 08536 USA

**Keywords:** Pomalidomide, IMiDs, Metabolism, Pharmacokinetics, Urinary excretion

## Abstract

**Purpose:**

To investigate the pharmacokinetics and disposition of [^14^C]pomalidomide following a single oral dose to healthy male subjects.

**Methods:**

Eight subjects were administered a single 2 mg oral suspension of [^14^C]pomalidomide. Blood (plasma), urine and feces were collected. Mass balance of radioactivity and the pharmacokinetics of radioactivity, pomalidomide and metabolites were determined. Metabolite profiling and characterization was performed. The enzymes involved in pomalidomide metabolism and the potential pharmacological activity of metabolites were evaluated in vitro.

**Results:**

Mean recovery was 88 %, with 73 and 15 % of the radioactive dose excreted in urine and feces, respectively, indicating good oral absorption. Mean *C*_max_, AUC_0−∞_ and *t*_max_ values for pomalidomide in plasma were 13 ng/mL, 189 ng*h/mL and 3.0 h. Radioactivity and pomalidomide were rapidly cleared from circulation, with terminal half-lives of 8.9 and 11.2 h. Pomalidomide accounted for 70 % of the circulating radioactivity, and no circulating metabolite was present at >10 % of parent compound. Pomalidomide was extensively metabolized prior to excretion, with excreted metabolites being similar to those observed in circulation. Clearance pathways included cytochrome P450-mediated hydroxylation with subsequent glucuronidation (43 % of the dose), glutarimide ring hydrolysis (25 %) and excretion of unchanged drug (10 %). 5-Hydroxy pomalidomide, the notable oxidative metabolite, was formed primarily via CYP1A2 and CYP3A4. The hydroxy metabolites and hydrolysis products were at least 26-fold less pharmacologically active than pomalidomide in vitro.

**Conclusions:**

Following oral administration, pomalidomide was well absorbed, with parent compound being the predominant circulating component. Pomalidomide was extensively metabolized prior to excretion, and metabolites were eliminated primarily in urine.

## Introduction

**Fig. 1:**
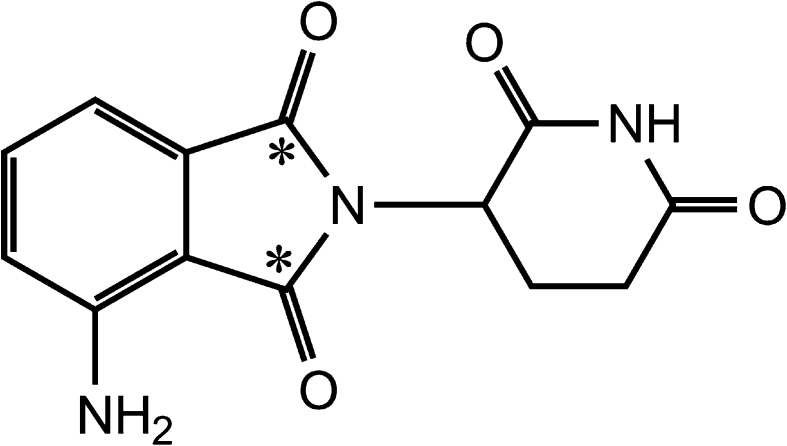
Structure of pomalidomide, with the site of the ^14^C label indicated (*)

Pomalidomide [CC-4047; (*RS*)-4-amino-2-(2,6-dioxo-piperidin-3-yl)-isoindoline-1,3-dione] (Fig. 1) is part of an immunomodulatory class of compounds that also includes thalidomide and lenalidomide. Compounds in this class are immunomodulators that can expand and activate T cells, natural killer cells and natural killer T cells as well as modulate the induction of pro- and anti-inflammatory cytokines, and recent in vitro studies have identified a major molecular mechanism for the pleiotropic effects of pomalidomide in multiple myeloma (MM) and in T cells [1]. Pomalidomide is under clinical development for the treatment of MM and myelofibrosis, as well as for the treatment of systemic sclerosis.

Pomalidomide is a racemic mixture of the S-enantiomer (CC-5083) and the R-enantiomer (CC-6016) which interconvert in plasma via both enzymatic and non-enzymatic pathways. The pharmacokinetics of pomalidomide in patients with relapsed or refractory MM following single or multiple (4 weeks) oral daily doses of 1, 2, 5 or 10 (single dose only) mg as capsules showed non-dose dependent absorption with a median *T*_max_ between 1.5 and 2.75 h following a single dose and between 2.9 and 4.0 h following multiple doses [2 and unpublished data]. Pomalidomide had a half-life of 6.5–8.0 h and showed little accumulation after once-daily administrations (mean *C*_max_ and AUC accumulation ratios <2). The current study was performed to evaluate the pharmacokinetics, metabolic disposition and excretion of a single oral suspension dose (2 mg, 100 μCi) of [^14^C]pomalidomide to healthy male subjects.

## Materials and methods

### Standards and reagents

[^14^C]Pomalidomide (Fig. 1) was prepared by Ricerca Biosciences, LLC (Concord, OH). The specific activity, radiochemical purity and chemical purity of the material were 49.15 μCi/mg, 99.6 % and 99.6 %, respectively. Reference standard for pomalidomide (>99.9 % chemical purity) was synthesized by Cambridge Major Laboratories, Inc. (Germantown, WI). Reference standards for CC-6016 (R-enantiomer of pomalidomide), CC-5083 (S-enantiomer of pomalidomide), CC-15262 (M10, hydrolysis product of pomalidomide), CC-8017 (M11, hydrolysis product of pomalidomide), CC-17369 (M16, 7-hydroxy pomalidomide), CC-17368 (M17, 5-hydroxy pomalidomide), CC-4067 (M18, *N-*acetyl pomalidomide), CC-12074 (M19, glutarimide ring hydroxylated pomalidomide), CC-17372 (6-hydroxy pomalidomide) and [^13^C_5_]-pomalidomide (internal standard) were synthesized by the Medicinal Chemistry and Process Chemistry groups at Celgene (Summit, NJ). 3-Aminophthalic acid (M2), thalidomide (analytical internal standard) and β-glucuronidase were obtained from Sigma-Aldrich (Milwaukee, WI). rCYP isozymes (Cypex Bactosomes) for reaction phenotyping were obtained from Cypex Ltd (Scotland, UK). All other reagents and chemicals were obtained from commercial sources.

### Clinical absorption, metabolism and elimination study

#### Study design and dose administration

This was an open-label, in-patient, single-dose study in eight non-smoking healthy male adult volunteers conducted in accordance with Good Clinical Practices (GCP) and in compliance with the Declaration of Helsinki. The protocol and consent form were reviewed and approved by the appropriate institutional review board, and all study participants gave written informed consent, before initiation of any study-related procedure. Male subjects were eligible based on standard inclusion/exclusion criteria and were excluded if they had exposure to excess radiation or participated in a study involving radioisotopes within the previous 12 months. Subjects entered the study center on the day prior to dosing and remained at the center for up to 15 days following dosing or until the total radioactivity recovered in excreta was ≥90 % or the radioactivity recovered in excreta per day on three consecutive days was ≤1 % of the administered radioactive dose.

Physical examination, clinical laboratory tests (hematology, serum chemistry and urinalysis), vital signs monitoring and 12-lead ECG were performed at the selected times before and after administration of the study drug. All subjects were monitored throughout the study for adverse events and for the use of concomitant medications.

Each subject was administered a single oral suspension of 2 mg (100 μCi) of [^14^C]pomalidomide in seltzer water (75 mL) followed by administration of an additional 140 mL of distilled water. Subjects were fasted for 8 h prior to and 4 h following dose administration. The residual radioactivity in the dosing vials was determined, and for six of the eight subjects (subjects 1–4, 6 and 8), the administered dose (1.93–2.02 mg) was close to the target dose of 2 mg. For subjects 5 and 7, a significant amount of radioactivity remained in the dose vial, resulting in administered doses of 1.59 and 1.51 mg, respectively. Calculations for percent of dose recovered in excreta were based on the actual administered doses.

Dose administration, sample collection, sample processing, and determination of total radioactivity were conducted at Covance Research (Madison, WI). Determination of pomalidomide in plasma, metabolite profiling and metabolite characterization were performed at XenoBiotic Laboratories, Inc. (Plainsboro, NJ).

#### Sample collection

Blood was collected into pre-chilled K_3_EDTA tubes at pre-dose, 0.5, 1, 1.5, 2, 2.5, 3, 4, 6, 8, 10, 12, 24, 48, 72, 120 and 168 h following dose administration. Blood for radioactivity counting was transferred to a clean tube and stored at or below 4 °C until analyzed. Plasma was harvested by centrifugation from the remaining blood. A plasma sample for radioactivity counting was transferred to a fresh tube. Three plasma aliquots were transferred to tubes containing citric acid for metabolite profiling and for determination of pomalidomide enantiomer concentrations in plasma. Urine was collected pre-dose, 0–6, 6-12, 12–24 and every 24 h thereafter up to 216 h (9 days) following dose administration and were stabilized with one volume of pH 1.5 Sorensen’s citrate buffer (25 mM sodium citrate buffer adjusted to pH 1.5). During the 12- to 24-h collection interval for subject 4, approximately 550 mL of urine was inadvertently poured into the wrong collection urinal and the sample was lost. The data presented do not account for this sample collection error, and the recovery for this subject represents partial dose recovered during that interval. Individual fecal samples were collected pre-dose and for up to 9 days following dose administration, and homogenized using approximately three volumes by weight of pH 1.5 Sorensen’s citrate buffer. All urine, fecal homogenates and plasma samples were stored at or below −20 °C until analyzed.

#### Radioanalysis

All radioactivity determinations were performed using Tri-Carb model 2900TR liquid scintillation counter (PerkinElmer, Wellesley, MA). For plasma, urine and residual dose vial analysis, duplicate samples were mixed with Ultima Gold XR scintillation cocktail and directly analyzed by liquid scintillation counting. For fecal homogenate and blood samples, duplicate aliquots were weighed, allowed to dry and combusted using a PerkinElmer model 307 sample oxidizer. The resultant [^14^C]CO_2_ was trapped in Carbosorb (PerkinElmer) in combination with Permafluor and assayed by liquid scintillation counting. For all matrices, any sample that was less than two times the background dpm was assigned a value of zero. Using these criteria, the lower limits of quantification in plasma, blood, urine and feces were 0.894 ng [^14^C]pomalidomide equivalent/mL (ngEq/mL), 0.939 ngEq/g, 0.871 ngEq/mL and 2.96 ngEq/g, respectively.

#### Measurement of pomalidomide enantiomers in plasma

Plasma concentrations of each pomalidomide enantiomer were determined using a chiral LC–MS/MS assay. The pomalidomide enantiomers and ^13^C-labeled pomalidomide (internal standard; IS) were extracted from plasma (stabilized with citric acid) using liquid–liquid extraction with methyl tertiary butyl ether. After transfer to a new tube, the solvent was evaporated, and the samples were reconstituted and injected for LC–MS/MS analysis using a Chiral AGP (50 × 3.0 mm, 5 μm; Chrom Tech, Inc., Apple Valley, MN) analytical column. Positive ions were measured in the multiple reaction monitoring (MRM) mode using a Sciex API-4000 tandem mass spectrometer equipped with a Turbo Ion Spray source. For the QC samples, the accuracy ranged from 91.0 to 106.9 %. For study samples, the amount of [^14^C]-labeled material was accounted for when calculating the concentration of enantiomers in plasma using an appropriate correction factor.

#### Metabolite profiling by HPLC

Individual plasma samples collected from all subjects at 1, 2.5, 4, 6, 10, 12 and 24 h post-dose were analyzed for metabolite profiling. Samples were extracted with acetonitrile, the supernatant was removed, and the pellet was washed with 0.1 % formic acid in water and extracted again with acetonitrile. The combined supernatants were adjusted to 15 or 25 mL by adding acetonitrile then evaporated to dryness under a stream of nitrogen. The residues were re-suspended in 0.1 % formic acid in water, assayed for extraction recovery and analyzed for metabolite profiling. A control plasma sample spiked with [^14^C]pomalidomide was extracted in a similar manner to determine the extraction efficiency and stability of [^14^C]pomalidomide.

For each subject, urine samples were pooled using an equal percentage by volume of the 0–6, 6–12, 12–24, 24–48 and 48–72 h samples. Pools of the 0–6, 6–12, 12–24, 24–48 and 48-72 h collections were also prepared across subjects and analyzed for metabolite profiling. Samples were mixed and centrifuged prior to the supernatants being transferred to fresh tubes for metabolite profiling analysis.

For each subject, fecal homogenates for metabolite profiling were pooled using an equal percentage by weight of the 0–24, 24–48, 48–72 and 72–96 h samples. Pools of the 0–24, 24-48, 48–72 and 72–96 h collections were also prepared across subjects and analyzed for metabolite profiling. An aliquot of each pool was extracted with acetonitrile; samples were mixed and centrifuged. The supernatant was removed and the pellets re-suspended with 0.1 % formic acid and extracted with acetonitrile. The combined supernatants were evaporated to dryness under a stream of nitrogen and the residue re-suspended in methanol:0.1 % formic acid in water (1:1). Samples were assayed for extraction recovery prior to metabolite profiling. A control fecal homogenate spiked with [^14^C]pomalidomide was extracted in a similar manner to determine the extraction efficiency and stability of [^14^C]pomalidomide.

HPLC analysis for metabolite profiling (HPLC Method 1) was performed using a Waters 2695 Alliance Separation Module (Waters Corp., Milford, MA) with the autosampler set to 4 °C. [^14^C]Pomalidomide and metabolites were separated using an ACE C18 column (150 × 4.6 mm, 3 μm; Advanced Chromatography Technologies, Aberdeen, Scotland) equipped with an ACE C18 guard column (10 × 3 mm, 3 μm). The mobile phases used were 25 mM ammonium acetate in water: pH 5.5 with formic acid (mobile phase A) and methanol (mobile phase B). The column temperature was 30 °C, and the flow rate was 0.7 mL/min. The linear gradient was delivered as follows: 0–2 min 100 % A; 2–38 min to 64 % A; 38–44 min to 0 % A; 44–48 min hold at 0 % A; return to initial conditions over 2 min.

Radioactivity profiles for plasma and excreta were determined by collecting the column eluate into 96-well Deepwell LumaPlates™ at a rate of 0.25 min/fraction. The plates were dried and the radioactivity in each well determined using a PerkinElmer TopCount^®^ NXT™ Microplate Scintillation Counter. HPLC radiochromatograms were reconstructed using LC-ARC data handling software.

For some urine and fecal extracts and for the isolation of metabolite M2 from urine, a subsample was subjected to solid-phase extraction and repeatedly injected and fractions collected in order to obtain sufficient metabolite concentrations to allow for mass spectrometry characterization. The M12 and M13 peaks isolated from urine were subjected to β-glucuronidase hydrolysis to confirm their identity. Isolated M12 or M13 in 0.1 M sodium phosphate buffer (pH 6.8) were incubated with β-glucuronidase (333 units) at 37 °C for 3 h. Samples were extracted with ethyl acetate, the supernatant was evaporated to dryness, and samples were re-suspended in acetonitrile:water (1:1) and analyzed by LC/MS.

#### Metabolite characterization by mass spectrometry

Selected plasma, urine and fecal extracts, as well as isolated metabolites, were analyzed by HPLC coupled with a radioactivity detector and mass spectrometer. For some analyses, mobile phase A was changed to 0.4 % formic acid in water, pH 3.2 adjusted with ammonium hydroxide, and mobile phase B was changed to acetonitrile (HPLC Method 2). Metabolites were characterized using a Thermo LTQ Orbitrap XL mass spectrometer (Thermo Scientific, Waltham, MA) or a SCIEX 4000 Q-Trap (Applied Biosystems, Foster City, CA), equipped with an electrospray source, operated in the positive ionization mode. The instrument settings and potentials were adjusted as necessary to provide optimal data. The Thermo LTQ Orbitrap mass spectrometer was operated with an ion spray voltage of 5.0 kV, a capillary temperature of 300 °C, a capillary voltage of 47 V and helium as the collision gas. For the SCIEX mass spectrometer, the electrospray needle potential was 5.0 kV; the turbo probe temperature was 450 °C, the curtain gas was nitrogen, the entrance potential was 10 V, the declustering potential was 60 V, and the collision energy was 20–30 eV.

#### Pharmacokinetic analysis

For pomalidomide and metabolite concentrations in plasma based on metabolite profiling data, pharmacokinetic data were generated by non-compartmental analysis of plasma versus time profiles using WinNonlin (version 4.1, Enterprise Edition).

Standard pharmacokinetic parameters were estimated from the pomalidomide (as the sum of the enantiomer concentrations at each time point) and enantiomer concentration versus time profiles or the blood and plasma radioactive concentration equivalents versus time profiles using established non-compartmental methods with WinNonlin (Version 5.2, Pharsight Corp., CA). Pre-dose samples that were below the limit of quantification (BLQ) or missing were assigned a numerical value of zero for the calculation of AUC. All post-dose BLQ were treated as missing in the PK analysis.

#### Calculations

The amount of total radioactivity (ngEq) in urine and feces was determined by multiplying the volume or weight of the samples by the radioactivity concentration. The dose recovered at each time point was determined by total radioactivity in the sample, divided by the total dose administered, multiplied by 100 %. For data BLQ values, a value of zero was assigned for calculations of means.

Total radioactivity in whole blood and plasma was converted to nanogram equivalent (ngEq) concentrations (ngEq/g for whole blood or ngEq/mL for plasma) based on the actual specific activity of the dose.

### In vitro CYP phenotyping

The CYP enzymes responsible for the formation of the hydroxy pomalidomide metabolites were investigated using 10 recombinant human CYP (rCYP) enzymes (CYP1A2, CYP2A6, CYP2B6, CYP2C8, CYP2C9, CYP2C19, CYP2D6, CYP2E1, CYP3A4 and CYP3A5) at 100 pmol/mL. Incubations were prepared using [^14^C]pomalidomide (1 μM) in 0.1 M potassium phosphate buffer (pH 7.4) with 4 mM MgCl_2_, pre-incubated for 5 min at 37 °C. Reactions were initiated by the addition of 1 mM NADPH and continued for 30 or 60 min. Reactions were quenched with two volumes of ice-cold 0.1 % formic acid in acetonitrile containing [^13^C_5_]pomalidomide as internal standard. The samples were mixed and centrifuged, and a subsample of the supernatant evaporated to dryness. The resulting pellet was reconstituted in 0.1 % formic acid in water and acetonitrile (4:1, v/v) and analyzed using HPLC with radiochromatography and/or LC–MS/MS. The rate of formation of the notable human metabolite M17 was calculated and scaled to human in vivo situation using relative abundance method [3].

### Pharmacological activity of pomalidomide metabolites

Metabolites and hydrolysis products of pomalidomide were tested for pharmacological activity in the following three assay systems: (1) inhibition of MM cell proliferation, (2) enhancement of primary human T cell IL-2 production and (3) inhibition of TNF-*α* production by lipopolysaccharide (LPS)-stimulated human peripheral blood mononuclear cells (PBMC). For all three assay types, compounds were dissolved in 100 % dimethyl sulfoxide (DMSO) and diluted into complete cell culture medium, consisting of Roswell Park Memorial Institute (RPMI)-1640 plus penicillin, streptomycin and l-glutamine (Mediatech, Manassas, VA). The MM cell proliferation cell culture medium contained 10 % fetal bovine serum, while the primary human cell cultures contained 10 % human AB serum (Invitrogen, Brown Deer, WI). The final DMSO concentration in the cell culture media was 0.25 %.

The human MM cell line OPM-2 was obtained from DSMZ (Braunschweig, Germany), and human MM cell lines U266 B1 and NCI-H929 were obtained from ATCC (Manassas, VA). Inhibition of MM cell proliferation was assessed by the [^3^H]-thymidine incorporation assay in a 3-day culture in the presence of compounds in complete medium, as previously described [4]. Primary human T cells were isolated from PBMC by negative selection using the RosetteSep T Cell Enrichment Cocktail (STEMCELL Technologies, Tukwila, WA) according to the manufacturer’s procedures. Costimulation of T cell IL-2 production was measured in a 2-day culture of primary human T cells isolated from healthy donors stimulated with anti-CD3 antibody in the presence of compounds, as previously described [4]. Inhibition of TNF-*α* production by LPS-stimulated human PBMC was measured in a 1-day culture in the presence of compound, as previously described [5].

## Results

### Clinical phase

All subjects were included in the pharmacokinetic, excretion and safety analyses. Subjects were between 21 and 31 years of age, with body mass index between 21.1 and 29.4 kg/m^2^. The race and ethnicity of the eight male subjects were 5 White, 2 American Indian/Alaskan Native (with one Hispanic/Latino) and 1 Indian.

#### Excretion of radioactivity

**Fig. 2:**
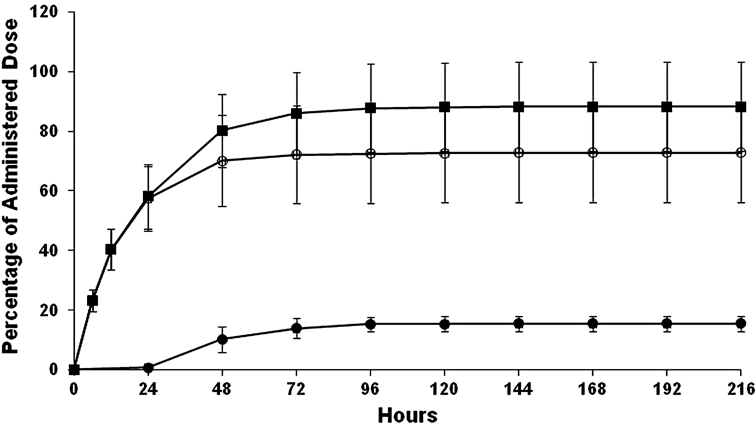
Cumulative elimination of radioactivity in urine and feces after a single oral 2 mg dose of [^14^C]pomalidomide in male healthy subjects (*open circle* urine, *filled circle* feces, *filled square* total); values are mean ± SD

Following a single 2 mg, 100 μCi dose of [^14^C]pomalidomide, urine and feces were collected from eight subjects over 216 h (9 days). Excretion of radioactivity ranged from 64.8 % to 110 %, with a mean total recovery of 88.3 %. For subject 4 (total recovery of 74.4 %), approximately 550 mL of urine collected during the 12–24 h collection was lost during sample collection and processing, resulting in a partial recovery for this subject during this interval. The amount of radioactivity (expressed as percentage of dose) excreted in urine ranged from 48.1 to 96.8 % (mean 72.8 ± 16.7 %), whereas fecal excretion ranged from 11.6 to 20.2 % (mean 15.5 ± 2.5 %) over the collection period. The majority of the radioactivity (>80 %) was recovered within the first 48 h after dose administration (Fig. 2).

#### Pharmacokinetic analysis

**Fig. 3:**
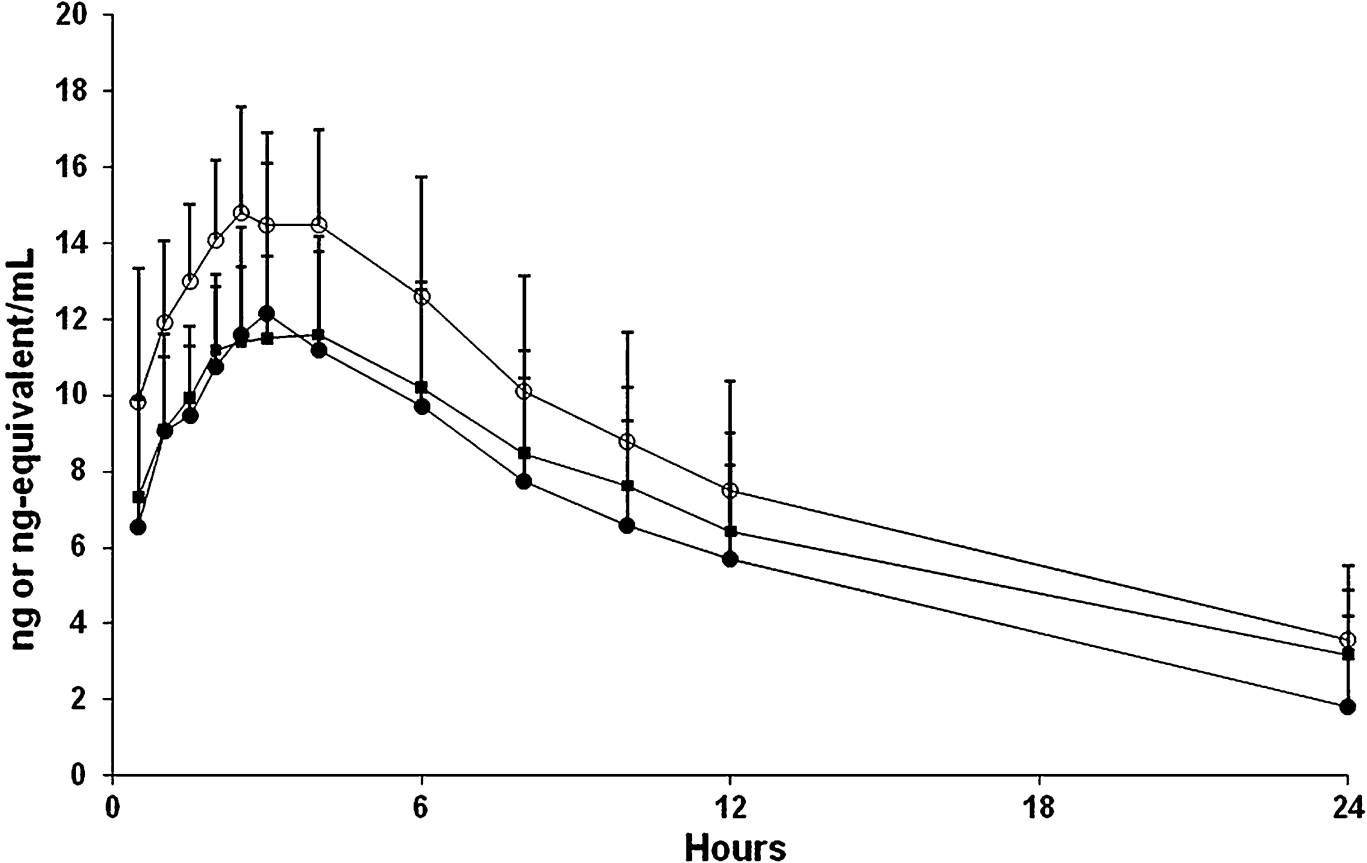
Concentration versus time curves for radioactivity in plasma (*open circle*), pomalidomide in plasma (*filled circle*), and radioactivity in blood (*filled square*) following a single oral 2 mg dose of [^14^C]pomalidomide in healthy male subjects; values are mean ± SD

**Fig. 4:**
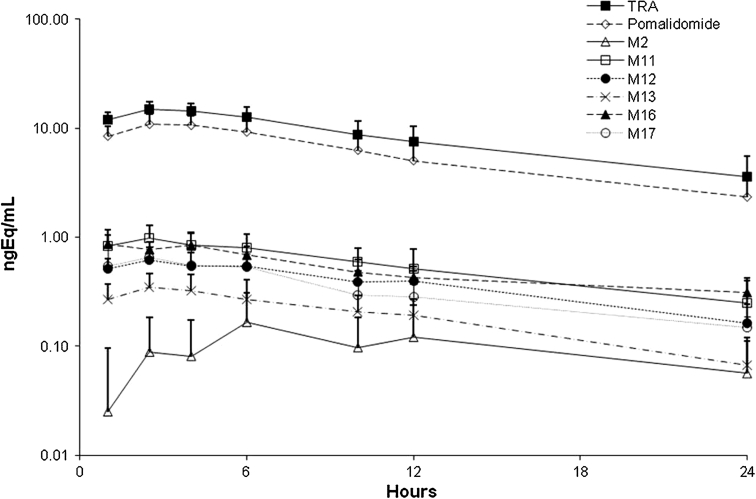
Concentration versus time curves for total radioactivity (TRA), pomalidomide, M2, M11, M12, M13, M16 and M17 in plasma following a single oral 2 mg dose of [^14^C]pomalidomide in healthy male subjects; values are mean ± SD

The absorption of [^14^C]pomalidomide was rapid (*T*_max_ of 3.0 h), and the plasma half-life of each pomalidomide enantiomer was 8.2–12.0 h (Table 1; Fig. 3). The *T*_max_ and half-lives for total radioactivity in plasma and blood were comparable to those for pomalidomide in plasma (Tables 1, 2), suggesting pomalidomide metabolites had similar elimination rates as parent compound. Pomalidomide was not detected in plasma beyond the 24-h time point, and radioactivity was not detected in blood or plasma past the 48-h collection. The pharmacokinetic parameters for pomalidomide determined via LC–MS/MS or via radiochromatography were comparable. The two pomalidomide enantiomers were present at similar levels in plasma and had similar pharmacokinetic parameters. Based on radiochromatography, plasma exposure (AUC) of pomalidomide was approximately 70 % relative to total radioactivity. The pharmacokinetic parameters of some of the more abundant metabolites were calculated using concentrations determined from radiochromatography (Table 2; Fig. 4). The peak plasma concentrations of the metabolites generally occurred at a similar time as pomalidomide (median *T*_max_ of 1–6 h for metabolites vs. 2.5 h for pomalidomide) and the metabolites had comparable mean *t*_½_ values relative to pomalidomide (9.1–14 h for metabolites vs 9.4 h for pomalidomide). Exposure (AUC) to these metabolites was between 1.7 and 6.3 % relative to total radioactivity. The mean blood/plasma ratios of radioactivity were between 0.75 and 0.90 in the samples collected up to 24 h post-dose (data not shown), indicating moderate distribution of radioactivity (pomalidomide and its metabolites) into the blood cells.

**Table 1:** Pomalidomide in plasma and radioactivity in plasma and whole blood pharmacokinetic parameters following a single oral 2 mg dose of [^14^C]pomalidomide in healthy male subjects

PK parameter	Pomalidomide in plasma^a,b^	S-enantiomer in plasma^b^	R-enantiomer in plasma^b^	Whole blood total radioactivity^c^	Plasma total radioactivity^c^	Radioactivity blood to plasma ratio
*C*_max_ (ng/mL)	13.0 ± 3.9	6.78 ± 1.99	6.36 ± 2.08	15.1 ± 2.7	12.1 ± 2.2	0.80
*T*_max_ (h)	3.0 (2.0–6.0)	3.0 (1.0–6.0)	3.0 (2.5–6.0)	2.5 (1.0–4.0)	3.25 (1.0–4.0)	NA
AUC_0−*t*_ (ng*h/mL)	137 ± 67	66.9 ± 34.1	67.7 ± 33.8	225 ± 99	189 ± 88	0.84
AUC_0−∞_ (ng*h/mL)	189 ± 52	96.4 ± 58.2	95.1 ± 46.1	260 ± 102	225 ± 94	0.87
*t*_½_ (h)	8.9 ± 3.4	8.8 ± 4.5	9.5 ± 3.2	10.2 ± 1.9	11.2 ± 2.5	NA

*NA* not applicable

^a^Values are reported as mean ± SD (*n* = 8) except *T*_max_ values, which are reported as median (min–max)

^b^Values determined using LC–MS/MS assay

^c^Units are ng [^14^C]pomalidomide equivalent/mL for radioactivity in blood or plasma

**Table 2:** Geometric mean (%CV) plasma pharmacokinetic parameters for pomalidomide and circulating metabolites after a single oral 2 mg dose of [^14^C]pomalidomide in healthy male subjects

	TRA	Pomalidomide^a^	M2	M11	M12	M13	M16	M17
*C*_max_ (ngEq/mL)	14.8 (17.8)	11.0 (22.5)	0.238 (36.3)	0.965 (33.3)	0.664 (23.5)	0.362 (31.7)	0.907 (27.4)	0.68 (33.3)
*T*_max_ (h)	2.5 (1.0–4.0)	2.5 (1.0–4.0)	6.0 (2.5–6.0)	2.5 (1.0–4.0)	2.5 (1.0–6.0)	3.25 (1.0–6.0)	1.0 (1.0–6.0)	2.5 (1.0–6.0)
*t*_½_ (h)	10.3 (23.5)	9.41 (25.0)	13.2 (10.7)	10.0 (30.0)	9.06 (18.7)	9.97 (43.6)	14.2 (30.2)	11.0 (20.8)
AUC_0–24_ (ngEq*h/mL)	194 (27.6)	135 (33.4)	3.31 (28.4)	12.3 (43.6)	8.90 (20.9)	3.86 (54.0)	11.9 (16.8)	7.76 (24.8)
AUC_0−∞_ (ngEq*h/mL)	245 (37.9)	165 (43.2)	4.27 (12.7)	15.9 (59.7)	10.1 (30.2)	5.99 (48.9)	17.6 (29.9)	11.5 (36.9)
AUC/AUC_TRA_ (%)	NA	69.6	1.71	6.34	4.59	1.99	6.13	4.00

*NA* not applicable, *T*_*max*_ median and range, *TRA* total radioactivity

^a^Pomalidomide and metabolite concentrations in plasma calculated using plasma radioactivity concentrations and radiochromatography

#### Metabolic profiles

**Fig. 5:**
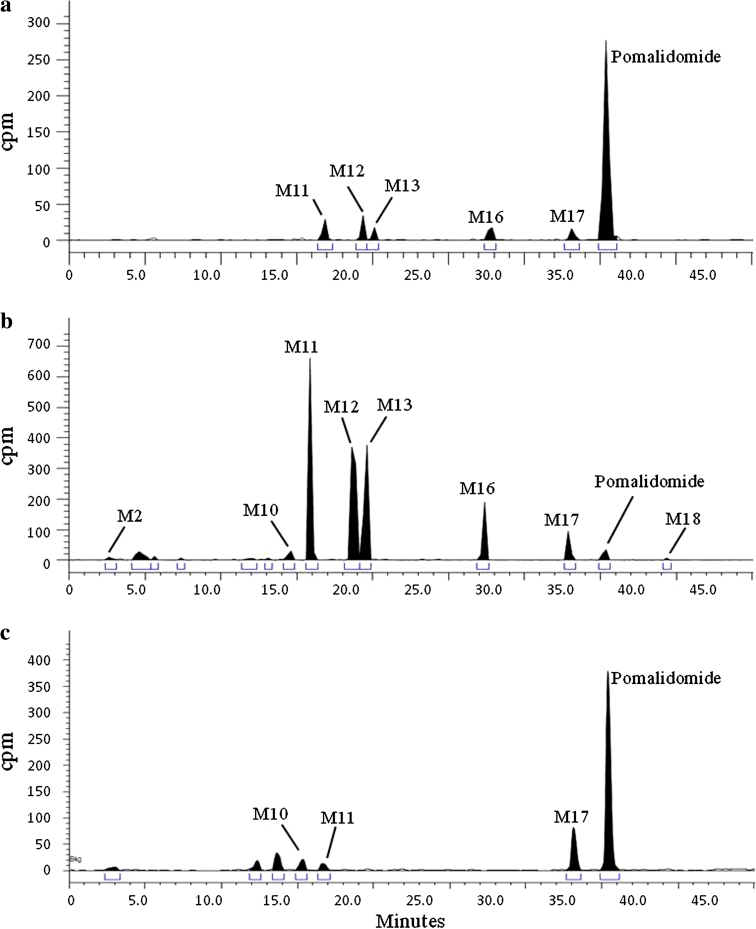
Representative radiochromatograms of **a** 2.5-h pooled plasma, **b** 0–72-h pooled urine, and **c** 0–96-h pooled feces after a single oral 2 mg dose of [^14^C]pomalidomide in healthy male subjects

HPLC radiochromatograms of plasma, urine and fecal homogenate from a representative subject are shown in Fig. 5. Metabolite profiles were similar between subjects and qualitatively similar at different time points. Pomalidomide was the predominant radioactive component in plasma. In plasma, pomalidomide plus metabolites M2, M11, M12, M13, M16 and M17 accounted for an average of 95.3 % of the radioactivity in the samples.

Pomalidomide was a minor component in urine, representing <3 % of the dose (Table 3). In urine, metabolites M11, M12 and M13 accounted for 23, 17 and 12 % of the dose, respectively, while metabolites M16 and M17 accounted for 7.0 and 3.2 % of the dose. At least eight additional radioactive peaks were observed in urine, each representing <2 % of the dose. In feces, pomalidomide represented 7.7 % of the dose, while the individual fecal metabolites each accounted for <3 % of the dose. In both urine and feces, <4 % of the dose was not characterized or identified as one of the metabolites listed in Table 3.

**Table 3:** Fragment ions and relative amounts for pomalidomide metabolites characterized in plasma, urine and feces

Metabolite characterization				Plasma (% AUC relative to TRA)^a^	% of dose excreted	
Name	Number	[M + H]^+^	Fragment ions		Urine	Feces
Pomalidomide	NA	274	246, 229, 201, 163, 84	69.6	2.2	7.7
3-Aminophthalic acid	M2	182	164, 120, 92^b^	1.7	1.4	ND
Hydrolysis product of pomalidomide	M10	292	275, 247, 229, 201	ND	1.5	0.3
Hydrolysis product of pomalidomide	M11	292	274, 247, 246, 229, 201, 163	6.3	23.3	0.3
Glucuronide conjugate of M17	M12	466	290, 262, 179, 84	4.6	17.1	ND
Glucuronide conjugate of M17	M13	466	290, 262, 179, 84	2.0	12.4	ND
7-hydroxy pomalidomide	M16	290	262, 245, 179, 84	6.1	7.0	ND
5-hydroxy pomalidomide	M17	290	262, 245, 179, 84	4.0	3.2	2.8
*N*-acetyl pomalidomide	M18	290	274, 246, 229, 201, 163, 84^b^	D	0.1	D
Glutarimide ring hydroxylated pomalidomide	M19	316	262, 245, 244, 217, 216, 189, 163^b^	D	D	ND

*ND* not detected, *D* detected by MS, but at concentrations too low to accurately quantify by radiochromatography

^a^AUC_0–24_ (unchanged drug or metabolite)/AUC_0–24_ (TRA) * 100 %

^b^Fragment ions are from authentic standard

#### Metabolite characterization

**Fig. 6:**
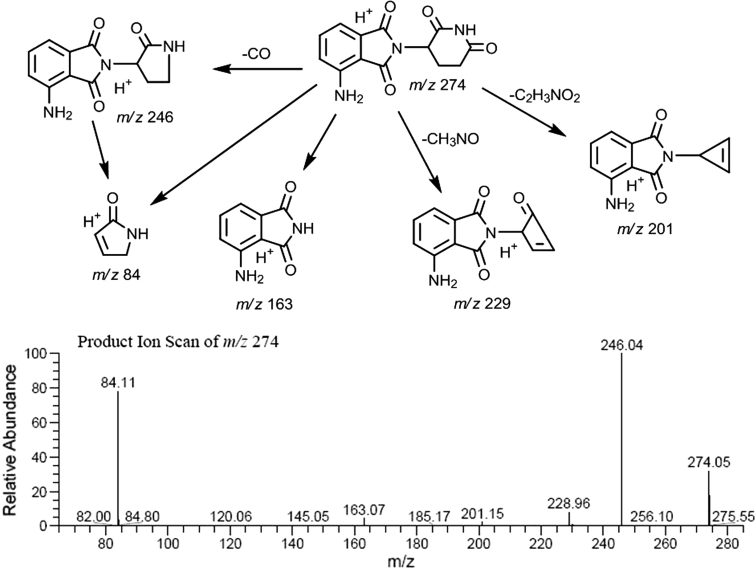
Mass spectral fragmentation of pomalidomide

**Fig. 7:**
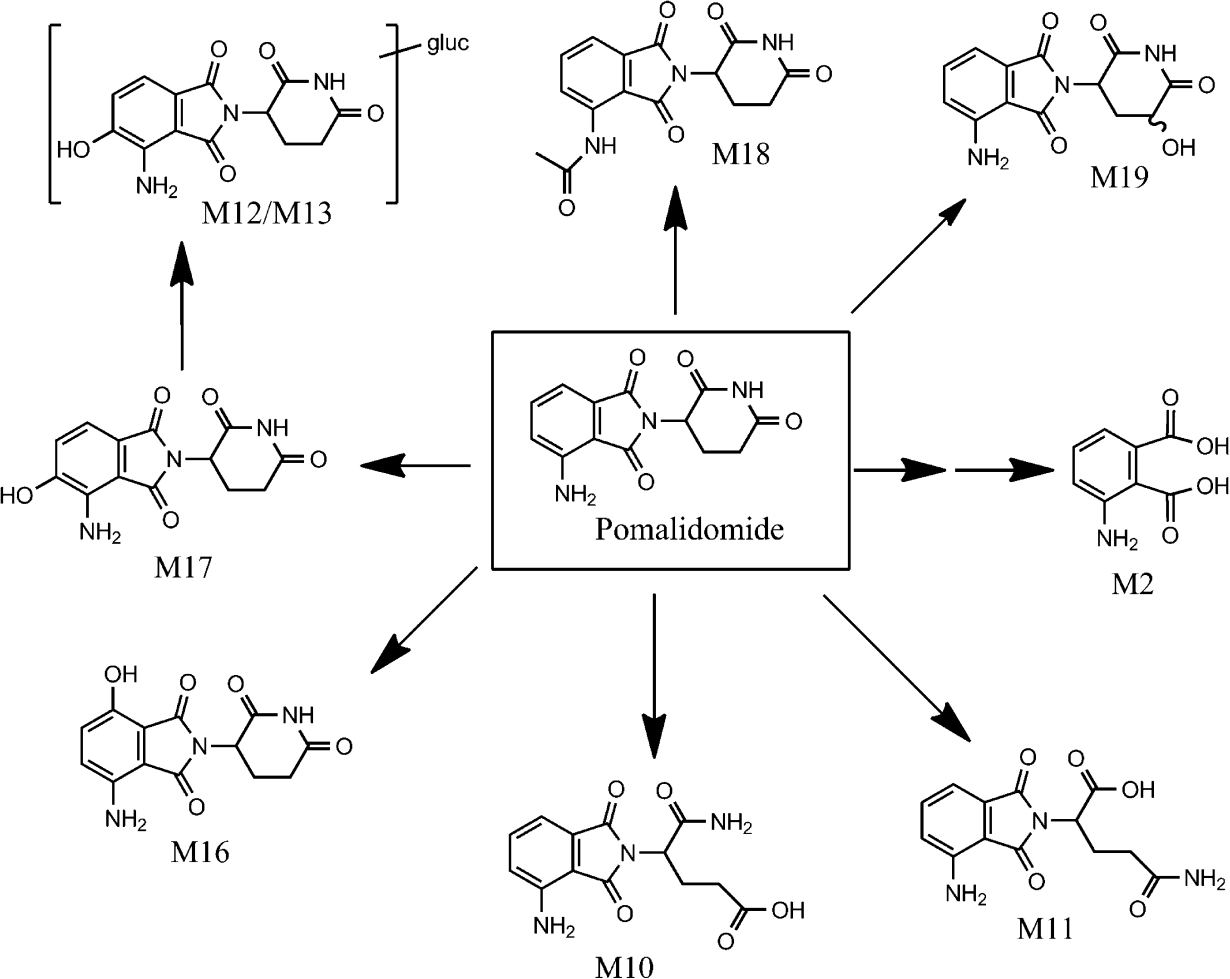
Metabolic scheme of pomalidomide in humans (*gluc* glucuronic acid)

Figure 6 shows the proposed mass spectrometry fragmentation pattern for pomalidomide, Table 3 lists the characteristic fragment ions for pomalidomide and the metabolites, and Fig. 7 shows the proposed metabolite structures. Using reference standard, pomalidomide generated a protonated molecular ion, [M + H]^+^, at *m/z* 274. The product ions of *m/z* 274 mass spectrum included *m/z* 246, 229, 201, 163 and 84. Loss of CO, CH_3_NO and C_2_H_3_NO_2_ generated *m/z* 246, 229 and 201, respectively. The aminoisoindoline-dione portion of the molecule generated *m/z* 163. Fragmentation of the piperidine dione to form a pyrrolone ring generated *m/z* 84.

Metabolite M2 was present at low levels and had an early retention time using HPLC Method 1, used for metabolite profiling. Therefore, in order to characterize metabolite M2, the radioactive peak was isolated from urine using HPLC Method 1, and the enriched sample was subjected to LC–MS/MS analysis using HPLC Method 2. Using this sample, metabolite M2 exhibited a characteristic mass ion peak corresponding to an [M + H]^+^ ion at *m/z* 182.0444, consistent with a molecular formula of C_8_H_8_O_4_N (−1.92 ppm). The retention time and accurate mass of metabolite M2 were consistent with the authentic standard of 3-aminophthalic acid. Thus, metabolite M2 was identified as 3-aminophthalic acid.

Metabolite M10 was present at low levels in urine and was isolated from urine for LC-MS/MS characterization. In the isolated sample, metabolite M10 had a [M + H]^+^ at *m/z* 292, which was 18 Da greater than pomalidomide, consistent with hydrolysis. Product ion *m/z* 201 for M10 was the same as for pomalidomide, suggesting the aminoisoindoline-dione portion of the molecule was intact. The fragmentation pattern and retention time of metabolite M10 were consistent with authentic standard for CC-15262, confirming M10 as a hydrolysis product of pomalidomide.

M11 exhibited a characteristic mass ion peak corresponding to an [M + H]^+^ ion at *m/z* 292.0927, consistent with a molecular formula of C_13_H_14_O_5_N_3_ (−0.35 ppm), indicating the addition of H_2_O to the parent molecule. Product ion *m/z* 201 for M11 was the same as for pomalidomide, suggesting the aminoisoindoline-dione portion of the molecule was intact. The fragmentation pattern and retention time of metabolite M11 were consistent with authentic standard for CC-8017, confirming M11 as a hydrolysis product of pomalidomide.

The [M + H]^+^ for both M12 and M13 in urine was observed at *m/z* 466, which was 192 Da greater than pomalidomide, consistent with hydroxylation plus glucuronidation. Both M12 and M13 exhibited characteristic mass ion peaks corresponding to an [M + H]^+^ ion at *m/z* 466.1092, consistent with a molecular formula of C_19_H_20_O_11_N_3_ (−0.01 ppm). Characteristic fragment ions for both M12 and M13 were observed at *m/z* 290 (aglycone), *m/z* 262 (aglycone loss of CO), *m/z* 179 (16 Da greater than the *m/z* 163 observed for pomalidomide) and *m/z* 84 (same as pomalidomide). Additionally, isolation of the M12 and M13 radioactive peaks followed hydrolysis by β-glucuronidase resulted in a single major radioactive aglycone product, which was identified by LC/MS to be metabolite M17 (5-hydroxy pomalidomide). Therefore, M12 and M13 were identified as glucuronide conjugates of 5-hydroxy pomalidomide.

The [M + H]^+^ for M16 was observed at *m/z* 290, which is 16 Da larger than pomalidomide, suggesting a hydroxylated metabolite. The product ions at *m/z* 262, 245 and 179 were 16 Da greater than the corresponding product ions for pomalidomide, suggesting the aminoisoindoline-dione portion of the molecule was the likely site of metabolism. The fragmentation pattern and retention time of metabolite M16 were consistent with authentic standard for CC-17369, confirming M16 as 7-hydroxy pomalidomide.

The [M + H]^+^ for M17 was observed at *m/z* 290, which is 16 Da larger than pomalidomide, suggesting a hydroxylated metabolite. The product ions at *m/z* 262, 245 and 179 were 16 Da greater than the corresponding product ions for pomalidomide, suggesting the aminoisoindoline-dione portion of the molecule was the likely site of metabolism. The fragmentation pattern and retention time of metabolite M17 were consistent with authentic standard for CC-17368, confirming M17 as 5-hydroxy pomalidomide.

Metabolite M18 had a retention time of 39 min in urine and was detected at very low levels using radiochromatography. Metabolite M18 had the same retention time as authentic standard CC-4067 (*N*-acetylated pomalidomide). The presence of M18 was confirmed in plasma, urine and feces with LC–MS/MS in the MRM mode using at least three fragment ions observed with authentic CC-4067. Based on retention time and MRM data, M18 was characterized as *N*-acetylated pomalidomide.

Metabolite M19 was not detected using radiochromatography. However, M19 was detected in trace amounts using LC–MS/MS in the MRM mode. Metabolite M19 had the same retention time as authentic standard CC-12074 (glutarimide ring hydroxylated pomalidomide). The presence of M19 was confirmed in plasma and urine with LC–MS/MS in the MRM mode using at least three fragment ions observed with authentic CC-12074. Based on retention time and MRM data, M19 was characterized as hydroxylated pomalidomide, with the site of hydroxylation on the glutarimide ring.

### CYP phenotyping

Using radiochromatography, metabolite M11 (hydrolyzed pomalidomide) was detected at low levels (2.5–5.9 %) in all rCYP incubations and in incubations performed in the absence of enzyme (5.5 %), suggesting that it is formed primarily via chemical degradation and/or non-CYP-mediated metabolism. No other metabolite peaks were observed by radiochromatography, so LC–MS/MS was used for subsequent analysis. Metabolite M16 (7-hydroxy pomalidomide) was not observed in any of the samples analyzed. Minor amounts of metabolite M17 (5-hydroxy pomalidomide) were observed in incubations with rCYP1A2, 2C19, 2D6 and 3A4, but were not detected with other rCYP isozymes or in the absence of enzyme. The rate of M17 formation following a 1-h incubation, based on peak area ratios, was low and estimated to be 0.0027, 0.0024, 0.0011 and 0.0008 pmol/min/pmol P450 following incubation with rCYP1A2, 2C19, 2D6 and 3A4, respectively.

Since the in vitro rate of metabolism was low, it was not possible to calculate a *K*_m_ or *V*_max_ values for the individual enzymes. Therefore, the relative contribution of each CYP isozyme to the total hepatic metabolism of pomalidomide was estimated based on the rate of metabolite formation for the individual isozymes and the relative abundance of each isozyme in human liver [3]. The relative abundance of the individual isozymes was obtained from Rowland-Yeo et al. [6].

### Pharmacological activity of pomalidomide metabolites

The hydrolysis products and phase I metabolites of pomalidomide were at least 26-fold less potent than pomalidomide for direct inhibition of MM cell proliferation (Table 4). Immunomodulatory activity (elevation of T cell IL-2 production and inhibition of peripheral blood mononuclear cell TNF-*α* production) of these compounds was at least 32-fold less potent than pomalidomide.

**Table 4:** Pharmacological activities of pomalidomide and its metabolites

Compound	OPM-2 MM cell proliferation IC_50_ (μM)	H929 MM cell proliferation IC_50_ (μM)	U266 MM cell proliferation IC_50_ (μM)	T cell IL-2 production EC_50_ (μM)	PBMC TNF-*α* production IC_50_ (μM)
Pomalidomide	0.038	0.035	0.028	0.022	0.0031
M10; pomalidomide hydrolysis product	>1	>1	>1	0.72	>10
M11; pomalidomide hydrolysis product	>1	>1	>1	>10	>10
M16; 7-hydroxy pomalidomide	>1	>1	>1	>10	>10
M17; 5-hydroxy pomalidomide	>1	>1	>1	>10	>10
M18; *N*-acetyl pomalidomide	>1	>1	>1	>10	>10
M19; glutarimide ring hydroxylated pomalidomide	>1	>1	>1	1.5	2.3

## Discussion

Pharmacokinetic analysis of total radioactivity and pomalidomide following a single oral [^14^C]pomalidomide dose to healthy volunteers resulted in plasma *T*_max_ values <3.5 h. Additionally, the amount of unchanged pomalidomide in feces (8 % of the dose), and the amount of radioactivity in urine (73 % of the dose), suggests absorption of the majority of the pomalidomide dose in humans. Elimination of radioactivity from blood and plasma, as well as pomalidomide and its metabolites from plasma, was similar with half-lives ranging between 9 and 14 h, indicating comparable clearance rates for all of the pomalidomide-related components. These data are consistent with the excretion data which showed recovery of radioactivity that was nearly complete and excretion was fairly rapid (>80 % radioactivity recovered at 48 h). Data from the current study are also consistent with pomalidomide pharmacokinetic data in patients with relapsed or refractory MM, which showed median *T*_max_ between 1.5 and 2.75 h and a half-life of 6.5–8.0 h [2 and unpublished data]. The pharmacokinetic profile of the R and S enantiomers of pomalidomide is comparable after the dosing of the racemate.

While pomalidomide was the predominant circulating component (approximately 70 % of the drug-related material in plasma), it was extensively metabolized prior to elimination in humans, with only 10 % of the dose excreted unchanged. The circulating and excreted metabolites were formed primarily via hydrolysis of the glutarimide ring (M10 and M11), hydroxylation of the phthalimide ring (M16 and M17) and subsequent glucuronidation (M12 and M13). Approximately 43 % of the pomalidomide dose was excreted as hydroxylated metabolites, primarily via formation of M17 (5-hydroxy pomalidomide), either with or without subsequent glucuronidation. The CYP phenotyping data indicate that several CYP isozymes are capable of metabolizing pomalidomide to M17. Accounting for the relative amount of each CYP isozyme in the liver, the relative contribution of each isozyme to the hepatic metabolism of pomalidomide was estimated to be 54, 11, 4 and 30 % for CYPs 1A2, 2C19, 2D6 and 3A4, respectively. These data indicate that CYP3A4 and CYP1A2 are the primary isozymes responsible for the CYP450-mediated metabolism of pomalidomide, with minor contributions from CYP2C19 and CYP2D6. However, individually none of these isozymes would be expected to contribute to >25 % of the overall pomalidomide elimination, when taking into account enzymatic and non-enzymatic metabolism, as well as elimination of parent compound.

Most of the circulating pomalidomide metabolites were tested for direct inhibition of MM cell proliferation and for immunomodulatory activity (elevation of T cell IL-2 production and inhibition of PBMC TNF-*α* production) and were at least 26-fold less active in these assays. In general, hydrolysis of the phthalimide ring, as in M10/M11, has been shown to decrease TNF-*α* inhibition [7]. Based on the current data, hydroxylation of the phthalimide ring also significantly decreases pharmacological activity.

The extent of drug-related material eliminated in urine is similar for pomalidomide (73 % of the dose) and the structurally related compounds thalidomide and lenalidomide (both >90 %). However, the composition of the drug-related material in urine for these three compounds is substantively different (Table 5). Pomalidomide is eliminated in urine primarily as metabolites and to a lesser extent as hydrolysis products. Thalidomide, which does not contain the aromatic amine group, is minimally excreted as parent compound, but instead is eliminated in the urine almost exclusively as glutarimide and phthalimide ring hydrolysis products [8], suggesting introduction of the amino group onto the phthalimide aryl ring makes pomalidomide more susceptible to oxidative metabolism. Furthermore, lenalidomide, which does not contain one of the phthalimide keto groups, has >80 % of the dose eliminated in urine as parent compound [9], suggesting this modification makes the compound more metabolically and chemically stable. Physiochemical properties such as ionization state, lipophilicity and polarity (polar surface area, relative polar surface area and hydrogen bond count) correlate with renal clearance [10]. Although these properties are predicted to be similar for these compounds based on chemical structures, the inherent stability and susceptibility to enzymatic hydroxylation may be the key factors in determining in what form these compounds are renally excreted.

**Table 5:** Urinary excretion profile for thalidomide, lenalidomide and pomalidomide

Compound	Structure	Percent dose excreted in human urine			
		Total	Parent	Hydrolysis products	Metabolites (oxidative and conjugative)
Thalidomide^a^		91.9	1.8	87.2	Trace
Lenalidomide^b^		90.3	81.7	ND	5.3
Pomalidomide		72.8	2.2	24.8	41.1

*ND* not detected

^a^Ref. [8]

^b^Ref. [9]

In summary, this study demonstrated that pomalidomide has good oral absorption (>70 %), is rapidly absorbed following oral dosing, and majority of the radioactivity is eliminated within 48 h. Pomalidomide was the predominant circulating component, and none of the metabolites represented >10 % of the plasma total radioactivity. However, pomalidomide represented only 10 % of the dose in excreta, with most of the remaining radioactivity eliminated as hydroxylated metabolites, glucuronide conjugates of these metabolites, as well as hydrolysis products of pomalidomide. Based on in vitro cell proliferation and TNF-*α* inhibitory effects of the metabolites and the relatively low systemic exposure to the metabolites, it is unlikely that metabolites contribute appreciably to the pharmacological activity of pomalidomide.
